# Predictors of the Therapeutic Response to Intralesional Bivalent HPV Vaccine in Wart Immunotherapy

**DOI:** 10.3390/vaccines9111280

**Published:** 2021-11-04

**Authors:** Noha M. Hammad, Ayman Marei, Gamal El-Didamony, Zeinb Mortada, Mona Elradi, Amira Hamed Mohamed Afifi, Heba M. Kadry

**Affiliations:** 1 Department of Medical Microbiology and Immunology, Faculty of Medicine, Zagazig University, Zagazig 44519, Egypt; ayman1067@hotmail.com (A.M.); hmshalapy@medicine.zu.edu.eg (H.M.K.); 2 Viral Infection Working Group of International Society of Antimicrobial Chemotherapy VIWG/ISAC, England and Wales, UK; 3 Department of Botany and Microbiology, Faculty of Science, Zagazig University, Zagazig 44519, Egypt; eldidamonyg@gmail.com (G.E.-D.); Hopema1900@hotmail.com (Z.M.); 4 Department of Dermatology, Venereology and Andrology, Faculty of Medicine, Zagazig University, Zagazig 44519, Egypt; mona.radi86@gmail.com; 5 Department of Clinical Pathology, Faculty of Medicine, Zagazig University, Zagazig 44519, Egypt; amira_hamed79@hotmail.com

**Keywords:** anogenital warts, bivalent HPV vaccine, cutaneous warts, human papillomavirus, interferon-gamma, interleukin-4, wart immunotherapy

## Abstract

Variable intralesional immunotherapies have recently been proposed as a means of achieving a successful eradication of recurrent and recalcitrant human papillomavirus (HPV)-induced cutaneous and anogenital warts. The bivalent HPV vaccine is one of the newly proposed immunotherapeutic agents. We investigated the role of interleukin-4 (IL-4) and interferon-gamma (IFN-γ) as ex vivo immunologic predictors to estimate the response to the bivalent HPV vaccine as a potential immunotherapy for cutaneous and anogenital warts. Heparinized blood samples were withdrawn from forty patients with multiple recurrent recalcitrant cutaneous and anogenital warts and forty matched healthy control subjects. Whole blood cultures were prepared with and without bivalent HPV vaccine stimulation. Culture supernatants were harvested and stored for IL-4 and IFN-γ measurements using an enzyme-linked immunosorbent assay. A comparative analysis of IL-4 and IFN-γ levels in culture supernatants revealed a non-significant change between the patient and control groups. The bivalent HPV vaccine stimulated cultures exhibited a non-significant reduction in IL-4 levels within both groups. IFN-γ was markedly induced in both groups in response to bivalent HPV vaccine stimulation. The bivalent HPV vaccine can give a sensitive IFN-γ immune response ex vivo, superior to IL-4 and sufficient to predict both the successful eradication of HPV infection and the ultimate clearance of cutaneous and anogenital warts when the bivalent HPV vaccine immunotherapy is applied.

## 1. Introduction

Human papillomavirus (HPV) is a small (50–55 nm in diameter) non-enveloped virus, possessing a circular double-stranded DNA genome which belongs to the family Papovaviridae, genus HPV. It exhibits tropism for epithelial cells, causing skin and mucous membrane infections in the form of cutaneous and genital warts [1]. The virus life cycle is entirely intraepithelial [2]. Viral entry to the basal keratinocytes possibly occurs via micro-abrasions in the surface epithelium, leaving the basal lamina of the skin intact [3].

The intraepithelial life cycle enables the virus to escape the host immune system by several mechanisms. Initially, the absence of inflammation during the virus replication process shuts down the danger signals required to alarm the innate immune system. HPV does not induce cell lysis because high-level virus replication and virus assembly occur in differentiated keratinocytes within terminal cutaneous layers destined to undergo apoptosis [4]. Furthermore, HPV gene expression and protein synthesis are restricted to keratinocytes; therefore, the processing of viral proteins does not happen in the professional antigen presenting cells (APCs) of the squamous epithelium. Additionally, there is no or only slight viremia. Virus shedding from the mucosal surface or skin occurs at a distance from blood vessels. Thus, it has poor access to the draining lymph node [2].

Most HPV infections can be spontaneously resolved within two years [5]. However, the cellular components of the immune system involved in this process are still being investigated. Early in infection, innate immune cells, including Langerhans cells (LC), dendritic cells (DC), natural killer (NK) cells, keratinocytes, and others are concerned with delivering an excellent adaptive immune response in the face of HPV. Most of these cells can mediate the release of proinflammatory mediators, linking innate and adaptive immune responses [6]. Moreover, NK cells can secrete IFN-γ and TNF-α and produce a direct cytotoxic effect against HPV, eliminating HPV-infected cells [5].

The balance between T helper 1 (Th1) and T helper 2 (Th2) cells might play a role in HPV infection. For years, the phenotypic dichotomy of activated T helper cells into Th1 and Th2 has been the essence of immunoregulation studies. IFN-γ, TNF-α, and IL-2 producing Th1 cells drive cellular responses, while IL-4-, IL-5-, IL-10-, and IL-13-generating Th2 cells incite humoral ones [7].

The proliferation of warts governed by the efficiency of cell-mediated immunity (CMI) demands novel prophylactic and therapeutic immunomodulatory options for those suffering from recurrent and resistant warts [8]. These options include (1) topical sensitizers, e.g., diphenylcyprone, (2) proinflammatory cytokines, e.g., interferons, and (3) intralesional antigen immunotherapies, e.g., Candida antigen, and the measles, mumps, and rubella (MMR), HPV, varicella-zoster virus (VZV), and Bacillus Calmette–Guérin (BCG) vaccines, and (4) others [9,10,11,12]. However, traditional approaches in managing warts are not convenient for multiple distant lesions. Moreover, the oppressive nature of these treatments and the high recurrence rate and the refractoriness of warts render immunomodulatory options more favorable and attainable [9,13,14].

Intralesional immunotherapy has become a convenient treatment, particularly for multiple recurrent and recalcitrant warts [15]. Despite the variable rates of complete response to different injected antigens [16,17,18], there is no definite cure. As a result, investigators always try to understand the underlying immune mechanisms when introducing a novel immunotherapeutic agent. Moreover, searching for immunologic predictors is mandatory to decide the best treatment option with favorable outcomes [19,20,21].

Cervarix is one of the prophylactic vaccines against HPV infection [22]. It is a bivalent vaccine that guards against high-risk HPV genotypes 16 and 18 and provides cross-protection against other oncogenic HPV genotypes such as HPV-31, 33, and 45 [23,24]. Moreover, IFN-γ is considered a pleiotropic molecule with associated antiproliferative, pro-apoptotic, and antitumor mechanisms [25]. However, in T cells, IL-4 induces the differentiation of naïve CD4 T cells into Th2 cells [26]. To date, there is not adequate evidence to demonstrate the potency of the prophylactic HPV vaccine in the treatment of HPV infections such as warts. Since IFN-γ and IL-4 are the signature cytokines of Th1 and Th2 immune responses, respectively, this work proposes investigating the significance of IFN-γ and IL-4 in predicting the therapeutic outcome when bivalent HPV vaccine immunotherapy is applied to patients with cutaneous and anogenital warts.

## 2. Materials and Methods

This study was carried out at the Immunology Research Laboratory, Microbiology and Immunology Department, Faculty of Medicine, Zagazig University.

### 2.1. Patients and Controls

Forty patients with recurrent and recalcitrant cutaneous and anogenital warts of different sites, sizes, and numbers were enrolled in the study by systematic random sampling from attendants in Zagazig University Hospital’s Dermatology outpatient clinic. Warts that were persistent for two years and failed to respond to two or more different treatments were considered recalcitrant [20]. In addition, all patients underwent complete history taking and clinical and dermatological examination. The study included forty age- and sex-matched healthy control subjects.

Exclusion criteria for this study were a history of allergy to the HPV vaccine, active viral, fungal, or bacterial infections, immunosuppressive diseases or drugs, autoimmune diseases, or other systemic diseases, e.g., hepatic or renal disorders, diabetes, meningitis or convulsions, skin allergies, pregnancy, lactation, or an earlier wart treatment at least one month before enrolment.

According to Nofal et al. [27], all patients initiated an intralesional injection (0.1 to 0.3 mL) of the bivalent HPV vaccine (Cervarix, GlaxoSmithKline, Brentford, UK), depending on the size of their largest wart. Patients received injections at two-week intervals until the complete elimination of their warts or until a maximum of six sessions had been completed. The evaluation of the therapeutic response depended on the contraction in the size and reduction in the number of warts [28]. The response was considered complete if there was a 100% clearance of warts, partial if there was a 50–99% reduction in wart size, and no response if there was less than a 50% reduction in wart size.

### 2.2. Blood Sampling

Three milliliters of fresh peripheral blood was collected from each study participant by venous puncture into sodium heparin containing vacutainers (10 U/mL). Blood was collected from patients before initiating bivalent HPV immunotherapy.

### 2.3. Preparation of Cultures

One milliliter of whole heparinized blood was cultured in 1 mL RPMI 1640 (Sigma-Aldrich, Missouri, United States) supplemented with L-glutamine, 100 U/mL penicillin, 100 μg/mL streptomycin (Invitrogen Cooperation, Grand Island, NY, USA), and 10% FCS. For each sample, three cultures were prepared. The diluted whole blood (WB) was incubated with the bivalent HPV vaccine at a concentration of 2.5 µL/mL for 48 h at 37 °C in humidified 5% CO_2_. Cultures stimulated with phytohemagglutinin-P (PHA-P: L9132 Sigma-Aldrich) at a 5 µg/mL concentration served as a positive control, while unstimulated cultures served as the negative control. After a 48 h incubation period, culture supernatants were harvested and preserved at −20 °C for cytokine measurement.

### 2.4. Measurement of IL-4 and IFN-γ

IL-4 and IFN-γ were estimated by a sandwich enzyme-linked immune sorbent assay (IL-4, Sunred, Shanghai, China, Catalog no. 201-12-0093, and IFN-γ: Invitrogen, CA, USA, Catalog no. KAC1231) according to the manufacturing company protocol.

### 2.5. Statistical Analysis

Statistical analysis was performed using Statistical Package for the Social Sciences software version 24 (SPSS version 24, Inc., Chicago, IL, USA). Data were expressed as mean ± standard deviation and median (quartile range) when appropriate. Parametric and nonparametric tests were used as appropriate. IL-4 and IFN-γ response change was calculated by dividing the cytokine level in stimulated cultures by the cytokine level in unstimulated ones. A receiver operating characteristic (ROC) curve analysis was conducted to identify the optimal IL-4 and IFN-γ responses to predict the therapeutic outcome. This method defined the optimal cut-point value whose sensitivity and specificity were closest to the value of the area under the ROC curve. *p*-values ≤ 0.05 were considered significant.

## 3. Results

The present study included two equal groups of forty patients and forty controls, matched in age and sex. The baseline features of the study participants are presented in Table 1.

### 3.1. Cytokine Measurement in Culture Supernatants

**Figure 1:**
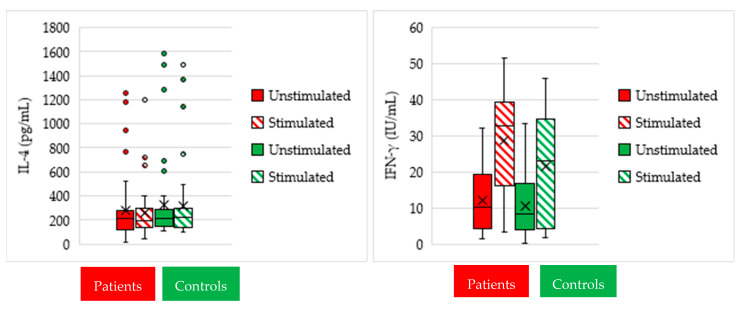
Boxplots showing the analysis of IL-4 (pg/mL) and IFN-γ (IU/mL) measurements (median and quartile range) in unstimulated and stimulated cultures in patient and control groups.

Comparative analysis of IL-4 (Figure 1) identified nonsignificant IL-4 change both between unstimulated (median: 212.6, and range: 15.9–1258.0 pg/mL) and stimulated (median: 197.1, and range: 47.5–1194.0 pg/mL) cultures within the patient group (*p* = 0.3), and between unstimulated (median: 214.0, and range: 109.2–1587.0 pg/mL) and stimulated cultures (median: 227.2, and range: 98.6–1486.0 pg/mL) within the control group (*p* = 0.8). Comparative analysis of IL-4 between the patient and control groups identified nonsignificant IL-4 differences in unstimulated and stimulated cultures (*p* = 0.47 and 0.48, respectively).

In the patient group, the median of IFN-γ was significantly elevated (*p* < 0.001) in stimulated cultures (median 32.6, and range: 3.4–51.6 IU/mL) when compared to unstimulated cultures (median 10.2, and range: 1.6–32.2 IU/mL). Within the control group, IFN-γ was significantly elevated (*p* < 0.001) in stimulated cultures (median 23.0, and range: 31.9–46.0 IU/mL) when compared to unstimulated cultures (median 8.2, and range: 0.25–33.3 IU/mL) as well. Comparison between the patient and control groups identified nonsignificant IFN-γ differences in unstimulated and stimulated cultures (*p* = 0.35 and *p* = 0.07, respectively) (Figure 1).

### 3.2. IFN-γ and IL-4 Responses as Predictors of Therapeutic Outcome

**Figure 2:**
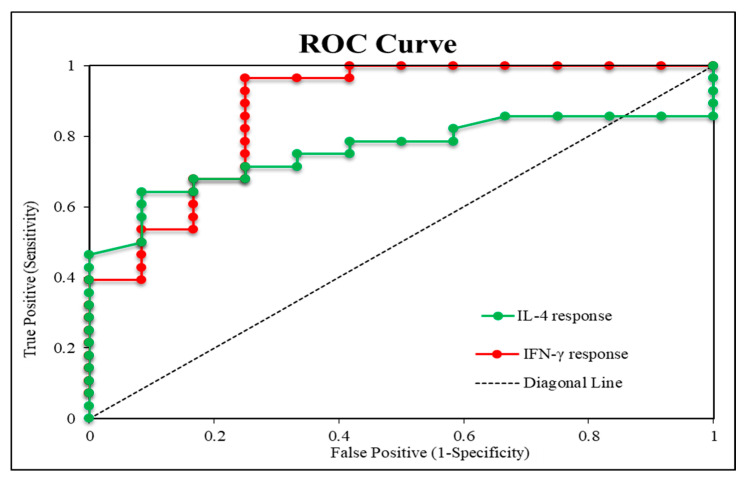
ROC (receiver operating characteristics) curve analysis demonstrating the validity of IFN-γ and IL-4 response in predicting the therapeutic response to immunotherapy in wart patients (*n* = 40).

The IFN-γ response, which was increased by ≥56.8% in the patient group, had 92.9% sensitivity, 75% specificity, and 87.5% accuracy. Conversely, the IL-4 response, which was reduced by ≥16.6%, had 62.2% sensitivity, 92.3% specificity, and 69.0% accuracy (Table 2 and Figure 2), indicating that IFN-γ is a reliable predictor for the therapeutic response to bivalent HPV vaccine immunotherapy compared to IL-4.

### 3.3. Correlation of IFN-γ and IL-4 Responses with Age in Patients

**Figure 3:**
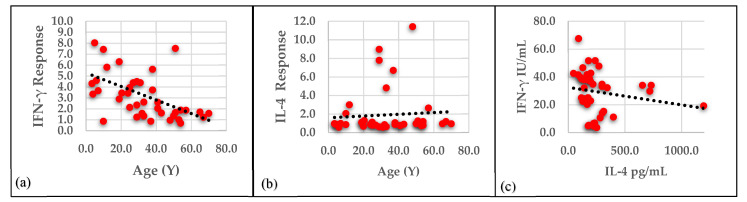
(**a**) Correlation between IFN-γ response and age in patient group. (**b**) Correlation between IL-4 response and age in patient group. (**c**) Correlation between IFN-γ (IU/mL) and IL-4 (pg/mL) level in stimulated cultures in the patient group (*n* = 40).

IFN-γ response had a significant inverse correlation with age (r = −0.6 and *p* < 0.001), while the correlation of IL-4 response with age was nonsignificant (r = 0.09 and *p* = 0.6). Stimulated cultures in the patient group revealed a significant inverse correlation between IFN-γ and IL-4 levels (r = −0.4 and *p* = 0.02) (Figure 3).

### 3.4. Correlation of Clinical Variables with Therapeutic Outcome

**Figure 4:**
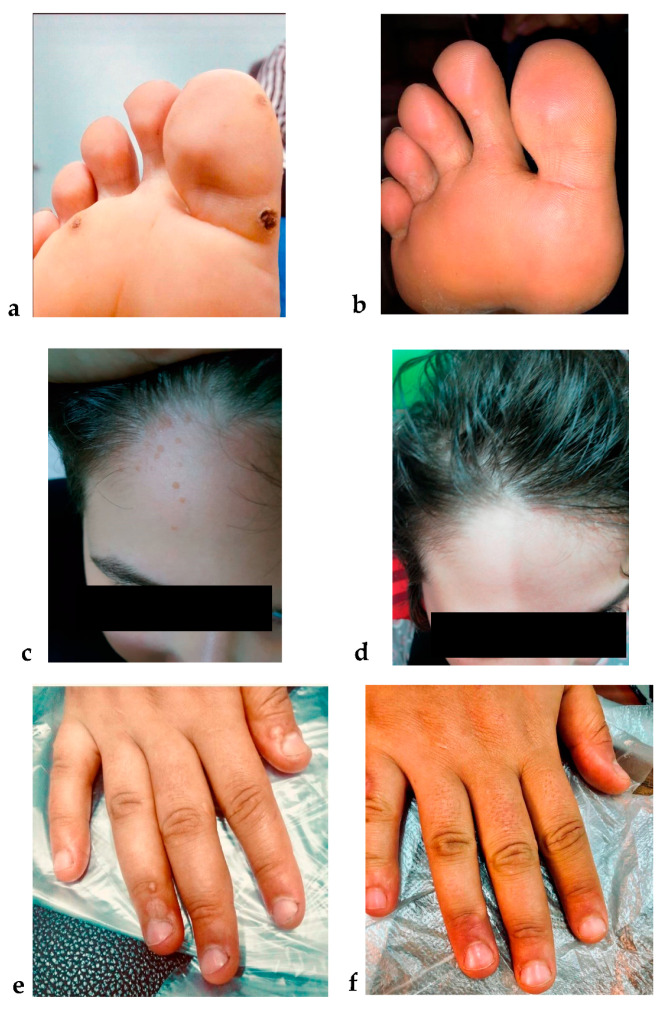
Complete response of multiple warts to bivalent HPV vaccine immunotherapy. Multiple plantar warts, (**a**) before and (**b**) after immunotherapy. Multiple warts on the face, (**c**) before and (**d**) after immunotherapy. Multiple warts on the dorsum of the hand, (**e**) before and (**f**) after immunotherapy.

In the present study, age, gender, recurrence, and recalcitrance did not affect bivalent HPV vaccine therapeutic response (*p* = 0.29, 0.08, 0.62, and 0.89, respectively). In addition, although the wart site did not influence the therapeutic outcome, all anogenital warts disappeared in 100% of their patients (*p* = 0.16) (Table 3 and Figure 4). Moreover, IFN-γ induction in stimulated cultures was significantly higher in responders when compared to partial and non-responders.

## 4. Discussion

Recent studies have reported the success of intralesional bivalent, quadrivalent, or nonavalent HPV vaccines in the treatment of recalcitrant warts [27,29,30]. However, to the best of our knowledge, this is the first study investigating the ex vivo role of the bivalent HPV vaccine in mounting an immune response in patients with cutaneous and anogenital warts. The eradication of HPV infection, and the resulting clearance of warts, mainly depends on CMI [31]. Mounting a successful cell-mediated immune response, especially the Th1 response, has always been the target of different immunotherapies.

In the present study, comparative analyses of WB cultures in the patient and control groups revealed a slight change in IL-4 levels in response to ex vivo stimulation by the bivalent HPV vaccine. On the other hand, a robust IFN-γ induction was observed within the patient and control groups, with minimal differences between the patient and control groups, indicating the immune system’s functionality.

Additionally, we observed a significant inverse relationship between IFN-γ and IL-4 responses. Moreover, when the patients received intralesional injections of the bivalent HPV vaccine and their therapeutic response was evaluated, the bivalent HPV vaccine immunotherapy achieved a complete response in 70% of the patients, which is comparable to what was reported by Nofal et al. (81%) [27]. Moreover, we observed a significant correlation between the IFN-γ response in cultures treated with the bivalent HPV vaccine and the therapeutic response; however, with IL-4, the correlation was nonsignificant. All these observations assume the mounting of a cell-mediated immune response with Th1 skewing in favor of Th2. Consistently, it is evident that successful intralesional antigen immunotherapy is associated with the Th1 cytokine profile—IL-2, IL-12, and IFN-γ—while the immunotherapy’s incompetence is linked to the Th2 cytokine profile—IL-4 and IL-10 [10].

Some authors reported the failure to associate baseline serum cytokines—IL-6, IL-10, TNF-α, and IFN-γ—with either the persistence or clearance of high-risk HPV infection [32]. Hence, we thought of exploring the production of IL-4 and IFN-γ in WB cultures stimulated by the bivalent HPV vaccine. Horn et al. [33] reported that intralesional immunotherapy induced the proliferation of peripheral blood mononuclear cells (PBMCs), releasing Th1, IFN-γ, which activates cytotoxic T cells and NK cells, with the eventual eradication of HPV infection. However, in the present study, we used WB as an alternative immunological assay instead. The WB assay is advantageous over PBMCs; it is rapid, needs no cell separation, and requires small blood volume. Moreover, it imitates the natural milieu of immunologic cells of both innate and adaptive immune systems rather than merely mononuclear cells (T cells and monocytes) [34]. The main disadvantage is that the total number of cells is unknown and cannot be controlled. However, we endeavored to overcome this obstacle by utilizing the calculated cytokine response change when appropriate.

We observed that IFN-γ response, unlike IL-4, significantly correlated with the therapeutic response in the present study. At a response of ≥ 56.8%, IFN-γ was able to predict the therapeutic response with 92.9% sensitivity, 75% specificity, and 87.5% accuracy. On the other hand, at a response of ≥ 16.6%, IL-4 was able to predict the therapeutic response with 62.2% sensitivity, 92.3% specificity, and 69% accuracy. Therefore, IFN-γ alone is superior to IL-4 and could be a reliable predictor for the therapeutic response to bivalent HPV vaccine immunotherapy. However, IL-4 could be a reliable predictor for the unfavorable outcome, particularly when coupled with IFN-γ.

In the present study, it is noteworthy that IFN-γ response on its own exhibited a significant indirect correlation with age, suggesting that the younger age group has a more robust immune response [33]. Nevertheless, the clinical variables including age, gender, recurrence, recalcitrance, and wart site did not correlate with the therapeutic outcome. Similarly, Kuan et al. [11], Yang et al. [35], and Nofal et al. [27] have reported that clinical variables did not affect the therapeutic outcome of the intralesional treatment of warts. However, the complete clearance of all anogenital warts in the studied patients was noteworthy in this study.

It is not surprising that natural HPV infection induces a modest humoral immune response due to poor shedding of the virus into blood vessels or lymphatics, and hence to lymph nodes where a humoral immune response is initiated [2]. However, seroconversion and type-specific antibodies to the major virus capsid protein L1 become detectable between 6 and 18 months in patients with persistent HPV infections [36]. However, the bivalent HPV vaccine can mount a successful long-term protective humoral immune response via the induction of antibodies with evident cross-protection [2].

Based on previous reports [11,27,35], we used the bivalent HPV vaccine instead of the quadrivalent HPV vaccine because it showed superior results in wart regression. The bivalent HPV vaccine achieved a complete response in 70% of the patients, which is comparable to Nofal et al. (81.8%) [27], though higher than Kuan et al. (26.9%) [11] and Yang et al. (46.67%) [35]. The mechanism by which HPV vaccines, including the bivalent vaccine, induce the regression of warts has not been unraveled yet. However, it has been suggested that the significant antigenic similarity of the L1 major capsid proteins of different HPV genotypes induces cross-protection with other nonvaccine genotypes [37]. Furthermore, the association of anogenital warts with high-risk genotypes targeted in the bivalent HPV vaccine is evident [38,39]. Additionally, the systemic response to the bivalent HPV vaccine exhibited elevated levels of IL-2, TNF-α, and proinflammatory cytokines (IL-1a, IL-1b, and IL-6), which could explain the clearance of warts [40].

Activated LCs secrete IL-12, which is essential for the activation and differentiation of naïve T cells into Th1 cells, the potent induction of IFN-γ secretion, and the differentiation and activation of cytotoxic T cells. In a previous study of anogenital warts treated with imiquimod immunotherapy, high levels of IFN-γ and IL-12 p40 mRNA in biopsies were reported. Moreover, the association of IFN-γ and IL-12 with clearance of warts in those patients was observed [41]. Moreover, the histopathological examination of regressing warts identified CD4+ and CD8+ T cells and macrophage infiltration into the wart stroma and epithelium with an expression of activation molecules on lymphocytes. The wart microenvironment is dominated by proinflammatory cytokines: TNF-α, IL-12, and IFN-γ. Consequently, wart capillaries upregulate the adhesion molecules necessary for the migration of lymphocytes. All these features favor the Th1 immune response [2].

Having a closer look at the composition of the bivalent HPV vaccine, we find that the bivalent vaccine consists of L1 virus-like particles (VLPs) of HPV 16 and 18 coupled to AS04, the trade name of a combination of adjuvants—aluminum hydroxide and monophosphoryl lipid A (MPL) [22]. AS04 was also observed to trigger APCs ex vivo. MPL, a detoxified derivative of lipopolysaccharide purified from Gram-negative bacteria, signals via TLR4, which in turn primes an innate immune response [42]. The stimulation of TLR4 activates the NF-κB signaling pathway, leading to the subsequent induction of proinflammatory mediators such as IL-6 and TNF-α. Consequently, these cytokines induce the maturation of Ag-loaded DCs and monocytes and suppress the tolerance response via the inhibition of T regulatory cells [43]. MPL promotes IFN-γ secretion by antigen-specific CD4+ T cells, shifting the immune response towards the Th1 profile [44]. On the other hand, the aluminum hydroxide component is responsible for the prolonged cytokine response to MPL. Therefore, the synergistic action of both components augments the bivalent HPV vaccine response both in vivo and ex vivo. Moreover, the VLPs in HPV vaccines are not infectious [45]; hence, they might be safely used in immunocompromised patients, unlike other types of immunotherapies.

## 5. Conclusions

IFN-γ is a pivotal cytokine responsible for inducing a robust Th1 immune response. Therefore, ex vivo quantitative analysis of IFN-γ is a reliable predictor for the appropriate therapeutic response to the promising bivalent HPV vaccine immunotherapy in wart patients, although IL-4 analysis could be a reliable predictor for the unfavorable outcome when combined with IFN-γ analysis.

## Figures and Tables

**Table 1:** Baseline characteristics of the study participants.

Variable	Patients *N* = 40	Controls *N* = 40	Test of Significance	*p*-Value
**Age (Year)**			*t*-test	0.14
Mean ± SD	32.8 ± 18.4	38.9 ± 18.3		
**Gender**			χ2 test	0.5
Female	20 (50.0)	17 (42.5)		
Male	20 (50.0)	23 (57.5)		
**Wart site**		N/A		
Face	7 (17.5)			
Dorsum of hands	14 (35.0)			
Dorsum of foot	2 (5.0)			
Plantar	10 (25.0)			
Back and axilla	1 (2.5)			
Genital	6 (15.0)			
**Recurrence**		N/A		
No	34 (85.0)			
Yes	6 (15.0)			
**Recalcitrance**		N/A		
No	17 (42.5)			
Yes	23 (57.5)			
**Response to IT**		N/A		
Responders	28 (70.0)			
Partial responders	4 (10.0)			
Non-responders	8 (20.0)			

SD, standard deviation; N/A, not applicable; IT, immunotherapy.

**Table 2:** Validity of the in vitro cytokine response to predict the therapeutic response the bivalent HPV vaccine.

Cytokine Response	Cutoff	AUC (95% CI)	*p*-Value	Sensitivity	Specificity	+PV	−PV	Accuracy
IFN-γ ↑	≥56.8%	0.88 (0.751–1.00)	*p <* 0.001	92.9%	75%	89.7%	81.8%	87.5%
IL-4 ↓	≥16.6%	0.688 (0.506–0.869)	*p =* 0.063	62.2%	92.3%	95.0%	51.2%	69.0%

PV; predictive value positive, −PV; predictive value negative.

**Table 3:** Relation between clinical variables, in vitro cytokine response, and therapeutic response to bivalent HPV vaccine.

Variable	Response (*N* = 40)			Test of Significance	*p*-Value
	Responders *n* = 28 (%)	Partial Responders *n* = 4 (%)	Non-Responders *n* = 8 (%)		
**Age (Years)**				One-way ANOVA	0.29
Mean ± SD	29.9 ± 16.8	43.3 ± 20.3	37.7 ± 22.6		
**Gender**				Fisher exact	0.08
Female	11 (39.3)	4 (100.0)	5 (62.5)		
Male	17 (60.7)	0 (0.0)	3 (37.5)		
**Wart Site**					0.016
Face	5 (17.9)	2 (50.0)	0 (0.0)		
Dorsum of hands	6 (21.4)	2 (50.0)	6 (75.0)		
Dorsum of foot	2 (7.1)	0 (0.0)	0 (25.0)		
Plantar	8 (28.6)	0 (0.0)	2 (25.0)		
Back and axilla	1 (3.6)	0 (0.0)	0 (0.0)		
Genital	6 (21.4)	0 (0.0)	0 (0.0)		
**Recurrence**					0.62
Yes	4 (14.3)	0 (0.0)	2 (25.0)		
No	24 (85.7)	4 (100.0)	6 (75.0)		
**Recalcitrance**					0.89
Yes	15 (53.6)	2 (50.0)	5 (62.5)		
No	13 (46.4)	2 (50.0)	3 (37.5)		
**IL-4 reduction response ^a^**				Kruskal–Wallis	0.14
Median	0.9	1.0	1.1		
Range	0.4–4.8	0.7–8.0	8.0–11.4		
**IFN-γ induction response ^a^**					0.001 *
Median	3.5	1.8	1.1		
Range	1.3–10.0	1.0–3.4	0.7–4.3		

IFN-γ, interferon gamma; IL-4, interleukin 4; ^a^ stimulated to unstimulated cytokine measurement ratio; * significant difference.

## Data Availability

The data of this study are available from the corresponding author on reasonable request.
